# Antiviral Drug Discovery for the Treatment of COVID-19 Infections

**DOI:** 10.3390/v14050961

**Published:** 2022-05-04

**Authors:** Teresa I. Ng, Ivan Correia, Jane Seagal, David A. DeGoey, Michael R. Schrimpf, David J. Hardee, Elizabeth L. Noey, Warren M. Kati

**Affiliations:** 1 Virology Drug Discovery, AbbVie Inc., North Chicago, IL 60064, USA; warren.kati@abbvie.com; 2 Department of Cell and Protein Sciences, Drug Discovery Science and Technology, AbbVie Inc., Worcester, MA 01605, USA; ivan.correia@abbvie.com; 3 Department of Biologics Discovery, Drug Discovery Science and Technology, AbbVie Inc., Worcester, MA 01605, USA; jseagal@alivamab.com; 4 Department of Centralized Medicinal Chemistry, Drug Discovery Science and Technology, AbbVie Inc., North Chicago, IL 60064, USA; david.degoey@abbvie.com (D.A.D.); michael.schrimpf@abbvie.com (M.R.S.); david.hardee@abbvie.com (D.J.H.); 5 Department of Structural Biology, Drug Discovery Science and Technology, AbbVie Inc., North Chicago, IL 60064, USA; elizabeth.noey@abbvie.com

**Keywords:** antiviral, COVID-19, SARS-CoV-2, drug discovery, coronavirus, spike protein, Mpro, RdRp, PLpro

## Abstract

The coronavirus disease 2019 (COVID-19) pandemic is caused by the severe acute respiratory syndrome coronavirus 2 (SARS-CoV-2), a recently emerged human coronavirus. COVID-19 vaccines have proven to be successful in protecting the vaccinated from infection, reducing the severity of disease, and deterring the transmission of infection. However, COVID-19 vaccination faces many challenges, such as the decline in vaccine-induced immunity over time, and the decrease in potency against some SARS-CoV-2 variants including the recently emerged Omicron variant, resulting in breakthrough infections. The challenges that COVID-19 vaccination is facing highlight the importance of the discovery of antivirals to serve as another means to tackle the pandemic. To date, neutralizing antibodies that block viral entry by targeting the viral spike protein make up the largest class of antivirals that has received US FDA emergency use authorization (EUA) for COVID-19 treatment. In addition to the spike protein, other key targets for the discovery of direct-acting antivirals include viral enzymes that are essential for SARS-CoV-2 replication, such as RNA-dependent RNA polymerase and proteases, as judged by US FDA approval for remdesivir, and EUA for Paxlovid (nirmatrelvir + ritonavir) for treating COVID-19 infections. This review presents an overview of the current status and future direction of antiviral drug discovery for treating SARS-CoV-2 infections, covering important antiviral targets such as the viral spike protein, non-structural protein (nsp) 3 papain-like protease, nsp5 main protease, and the nsp12/nsp7/nsp8 RNA-dependent RNA polymerase complex.

## 1. Introduction

Severe acute respiratory syndrome coronavirus 2 (SARS-CoV-2), a novel human coronavirus that emerged in late 2019, is the etiological agent of coronavirus disease 2019 (COVID-19) [1,2]. The COVID-19 pandemic has presented unprecedented challenges to health care, economics, and societies globally. At the time of writing (February of 2022), SARS-CoV-2 infection has exceeded 400 million cases, resulting in approximately 5.8 million deaths worldwide [3]. SARS-CoV-2 is a member of the betacoronavirus genus, the same genus as the two highly pathogenic human betacoronaviruses known as SARS-CoV and MERS-CoV, which were responsible for the deadly outbreaks in 2002 and 2012, respectively [4,5].

### 1.1. Medical Countermeasures for COVID-19

To combat the COVID-19 pandemic, there has been immense effort in the discovery and development of medical countermeasures including vaccines and drug treatments for COVID-19. COVID-19 vaccine development achieved scientific breakthroughs, delivering several vaccine candidates that received the US Food and Drug Administration (FDA) emergency use authorization (EUA) in less than a year after the start of the COVID-19 pandemic. COVID-19 vaccines have significantly reduced morbidity and mortality in the vaccinated population [6]. However, lessons learned from the use of COVID-19 vaccines since the first vaccine approval in December 2020 indicate that COVID-19 vaccination alone cannot effectively address the current pandemic, and additional treatment options are required to end this global challenge. COVID-19 vaccination faces many hurdles to control COVID-19 infections worldwide: limited availability that results in inequality in global access, logistic challenges to distribute vaccines requiring special storage conditions to remote areas, a requirement for 2 doses for some vaccines to establish satisfactory immune-protection [7], waning immunity in as little as 6 months after completion of the initial vaccine administration thus requiring boosting [8,9,10], and emergence of SARS-CoV-2 variants resistant to the immunity induced by vaccines [11,12,13,14]. Breakthrough infections in vaccinated populations with the recently emerged SARS-CoV-2 Omicron variant exposed some of these limitations of COVID-19 vaccines and highlight the need for other medical treatments such as drug therapy, especially those that are broad spectrum and can be administered orally, to complement the use of vaccines. The strategies for COVID-19 drug discovery can be divided into two categories, targeting host factors or viral proteins that are important for the life cycle and/or pathogenesis of SARS-CoV-2 infections. This review focuses on the discovery of COVID-19 drugs that directly act against viral proteins. Direct-acting antiviral therapeutics have a good track record for treating viral diseases, such as those caused by human immunodeficiency virus (HIV), hepatitis C virus (HCV), hepatitis B virus (HBV), herpesviruses, and influenza virus. In addition, some of the recently developed COVID-19 direct-acting antivirals have also demonstrated efficacy in clinical settings. 

### 1.2. Representative Viral Targets for COVID-19 Antiviral Intervention

Several SARS-CoV-2-encoded proteins have been identified as promising molecular targets for antiviral intervention due to their essential roles in the viral life cycle [15,16,17]. The entry of SARS-CoV-2 is mediated by the binding of the viral spike (S) protein to the host cell receptor angiotensin-converting enzyme 2 (ACE2) [18,19]. After entry, SARS-CoV-2 viral RNA is translated by the host to produce two polyproteins from two overlapping open reading frames (ORFs), ORF1a and ORF1b. The polyproteins are then proteolytically cleaved by two virally encoded cysteine proteases, the non-structural protein (nsp) 3 papain-like protease (PLpro) and the nsp5 main protease (Mpro, also known as 3CLpro) to yield 16 individual nsps [20]. A subset of these nsps associate to form a replication–transcription complex that mediates RNA synthesis, capping and proofreading. The nsp12 RNA-dependent RNA polymerase (RdRp) is a key viral enzyme that mediates viral replication and transcription. In short, the S protein, PLpro, Mpro, and RdRp represent prime targets for SARS-CoV-2 antiviral drug discovery. Not surprisingly, SARS-CoV-2 antivirals that have received US FDA formal approval or EUA for COVID-19 treatment encompass inhibitors targeting many of these viral proteins (Table 1).

### 1.3. Strategies of Antiviral Drug Discovery for COVID-19

Different strategies have been used in antiviral drug discovery for COVID-19, such as drug repurposing of approved or investigational drugs beyond their original indications, high-throughput screening, computer-aided virtual screening, and structure-based drug discovery. At the onset of the COVID-19 pandemic, there was great interest in drug repurposing as an expedited means to identify COVID-19 drugs [21,22]. There was indeed some success in drug repurposing for COVID-19 treatment. Remdesivir (RDV), which was originally developed for the treatment of Ebola virus infection, was found to be active against SARS-CoV-2 [23,24] and has successfully been developed into a COVID-19 drug [25]. Molnupiravir was originally discovered for Venezuelan equine encephalitis virus (VEEV) infection, but was later found to have antiviral activity against a number of respiratory viruses, including influenza and, most recently, SARS-CoV-2 [26,27]. When the COVID-19 pandemic started, the development of molnupiravir was quickly switched from influenza to COVID-19, and it received US FDA EUA to treat COVID-19 infections in December 2021 [26,27]. In addition to drug repurposing, novel molecules including a number of anti-S neutralizing monoclonal antibodies (mAbs) and an Mpro inhibitor have also been discovered and developed as COVID-19 drugs (Table 1). Antiviral drugs used for the treatment of COVID-19 infections provide protection from infection or improvement in recovery, but all COVID-19 antivirals available to date have some limitations that may make them not suitable for use in the general population. For example, molnupiravir is not recommended for use in pregnancy because it may cause fetal harm [28]. Another example is Paxlovid (ritonavir-boosted nirmatrelvir), which has the potential for complex drug–drug interactions with concomitant medications because (1) ritonavir is a CYP3A inhibitor, and may therefore increase plasma concentrations of drugs that are predominantly metabolized by CYP3A, and (2) ritonavir and nirmatrelvir are substrates of CYP3A; drugs that induce CYP3A may thus decrease ritonavir and nirmatrelvir drug levels in plasma, reducing Paxlovid therapeutic potency [29,30]. Drug resistance of emerging SARS-CoV-2 variants is also a concern as exemplified by the action of the FDA in January 2022 to limit the use of the anti-S mAb cocktail of bamlanivimab and etesevimab, as well as the mAb cocktail of casirivimab and imdevimab in COVID-19 patients, as both of these mAb cocktails are not active against the Omicron variant, which was circulating in the US at a very high frequency at that time [31]. Furthermore, patients receiving COVID-19 drugs that require IV administration (e.g., most anti-S mAbs and RDV) need access to medical facilities capable of delivering these drugs. Due to these limitations, there is an urgent need to discover novel and improved antiviral drugs with convenience of administration, improved safety and drug property profiles, and broad-spectrum coronavirus coverage to combat the current COVID-19 crisis as well as emerging coronaviruses with spillover and pandemic risk. This review provides an up-to-date overview on the discovery of antiviral drugs for COVID-19, covering the functions of important antiviral targets such as the viral S protein, Mpro, RdRp, and PLpro, and the different inhibitors against these targets.

## 2. Spike Protein (S Protein)

Coronaviruses are enveloped viruses and their successful entry into the host target cells requires completion of two steps [32,33,34]. The first is binding to a host cell receptor and the second is fusion of the viral envelope with the host cell membrane, which releases viral genome into the cytoplasm, enabling viral replication. Both steps are mediated by the S protein, a heavily glycosylated class I fusion protein that is present on the envelope of the virus as a trimer [35]. The importance of the viral S protein to host cell entry makes it an ideal target for antibody (Ab)-based therapeutics [36,37,38].

### 2.1. Structural Organization of Spike

**Figure 1:**
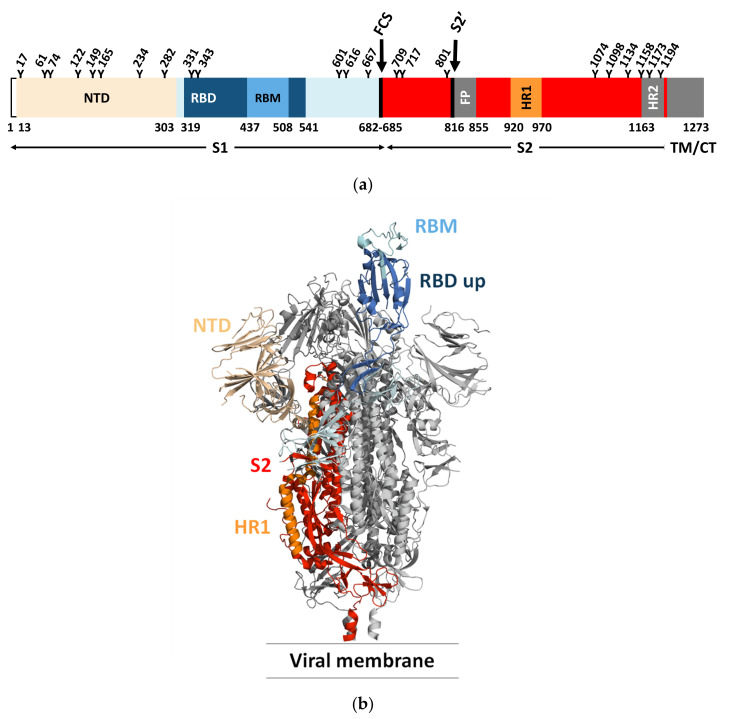
(**a**) Domain architecture of the SARS-CoV-2 spike protein, comprising the N-terminal domain (NTD), the receptor-binding domain (RBD), the receptor-binding motif (RBM), the furin cleavage site (FCS), the S2′ cleavage site, the fusion peptide (FP), and heptad repeats 1 and 2 (HR1 and HR2), as they relate to the S1 and S2 subunits, as well as the transmembrane domain (TM) and the cytoplasmic tail (CT). Glycosylation sites are marked at the top of the figure. (**b**) Side view of the pre-fusion structure of the SARS-CoV-2 spike protein (PDB ID 6VYB [39]) with a single RBD in the “up” state and exposing the RBM. The two RBD “down” protomers are shown in gray and the RBD “up” protomer is shown in color corresponding to the schematic in (**a**).

Each monomer in the S trimer consists of the receptor-binding S1 and a membrane-anchored S2 subunit that contains the membrane fusion machinery (Figure 1a) [19,32,34,39,40]. The receptor-binding S1 subunit has an N-terminal domain (NTD) and a receptor-binding domain (RBD), also called the C-terminal binding domain (CTD). The RBD is composed of a core region and a receptor-binding motif (RBM) that the virus uses to interact with the host cell receptor. The RBM of the S protein is prone to mutations, while the core region is more conserved [41]. The membrane-anchored S2 subunit has the fusion peptide (FP), a domain rich in hydrophobic residues, which is inserted into the host cell membrane. The S2 domain also contains two heptad repeats, HR1 and HR2, that form a six-helix bundle to complete the fusion process and delivery of the viral genome into the cytoplasm [40]. Like many other viral fusion proteins, the SARS-CoV-2 S protein is covered by a glycan shield protecting the S protein from host immune recognition. Both SARS-CoV and SARS-CoV-2 S proteins present a different glycosylation pattern from that of the HIV-1 envelope protein, showing a larger presence of complex N-glycans relative to oligomannose type [35]. The SARS-CoV-2 S protein has 22 predicted N-glycosylation sites per protomer plus at least two predicted O-glycosylation sites. The RBD is less protected by glycans and is therefore more immunogenic. Natural Ab responses are mostly directed toward the RBD and, as described further below, many mutations arise in this domain to escape Ab neutralization [42].

### 2.2. Interaction of Spike with Receptor and Mechanism of Viral Entry

SARS-CoV-2 and SARS-CoV share the same host cell receptor, ACE2, whereas for MERS-CoV, the receptor is dipeptidyl peptidase 4 (DPP4) [43,44,45]. The RBM has a high tolerance for mutations and both SARS-CoV and SARS-CoV-2 bind to the receptor in a similar manner despite low sequence similarity. In addition to depending on ACE2 for host cell entry, both SARS-CoV and SARS-CoV-2 depend on entry activation by host cell proteases (cathepsin L, TMPRSS2) acting at the S1/S2 boundary and an S2 site upstream of the fusion peptide called S2′ of the S protein (Figure 1a) [46]. The SARS-CoV-2 S protein also has a furin cleavable site that potentiates SARS-CoV-2 infectivity (Figure 1a) [47]. Of interest, although the interaction of the S protein with relevant host receptor plays a critical role in tissue tropism, other “background genes”, including nucleocapsid and replicase as well as accessory genes, may also impact tissue tropism [48]. 

Cryo-EM and single-particle reconstruction have provided structural information on the trimeric S protein [34,49]. In the closed pre-fusion conformation, all three RBDs lie flat (“down” state) and make the RBMs inaccessible for biological interactions, while in the open pre-fusion conformation, one or more RBDs are lifted (“up” state) to expose the corresponding RBMs, enabling S protein/ACE2 interactions (Figure 1b) [49].

**Figure 2:**
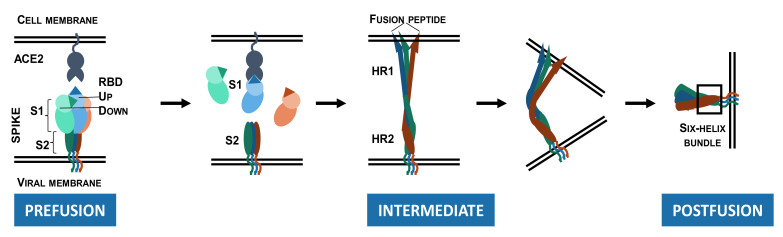
Conformational changes in the SARS-CoV-2 spike ectodomain during membrane fusion. Pre-fusion conformation: Spike protein with two RBDs in the “down” state and one RBD in the “up” state with the RBM exposed and available for binding to the ACE2 receptor. The spike protein/ACE2 interactions induce shedding of the S1 subunits. Intermediate conformation: The S2 subunits become elongated and reach out to the host cell membrane, enabling insertion of the fusion peptide. Post-fusion conformation: HR2 forms a six-helix bundle with HR1 inducing fusion of the viral membrane with the host cell membrane.

As depicted in Figure 2, S protein binding to ACE2 destabilizes the pre-fusion trimer, resulting in shedding of the S1 subunit, and significant conformational change in the membrane-bound S2 subunit. The S2 subunit acquires an elongated shape, enabling the insertion of the fusion peptide into the host cell membrane (intermediate conformation). The intermediate conformation is unstable and rapidly transitions to a stable post-fusion conformation in which HR1 and HR2 form a six-helix bundle. During this transition, viral and host-cell membranes are brought to a close proximity, resulting in the membrane fusion [34,49,50].

### 2.3. Anti-SARS-CoV-2 Antibodies Recognizing the Spike RBD

Monoclonal antibodies that target SARS-CoV-2 have been isolated from convalescent COVID-19 patients, SARS-CoV patients, as well as immunized wild-type and transgenic mice. Three-dimensional structures are available for mAbs either in complex with the ECD or the RBD of the S protein, and most mAbs target immunodominant epitopes in the RBM though several of them target the core RBD, the NTD and the S2 subunit [51,52,53].

Antibodies targeting the RBD of the S protein can be assigned to four functional classes based on binding epitope determined by cryo-EM or high-resolution X-ray crystallography [54]. Class 1 and 2 Abs directly block ACE2, whereas class 3 and 4 do not; class 1 and 4 only bind to the “up” RBDs, whereas class 2 and 3 bind to RBDs regardless of their “up“ and “down“ states [33,55].

- Class 1 mAbs are ACE2 blocking and only bind to the “up” RBDs and prevent viral entry into the cell. Many are VH3-53 or VH3-66 Abs. Examples include etesevimab (LY-CoV016), casirivimab (REGN10933) and tixagevimab (AZD8895).
- Class 2 mAbs are ACE2 blocking and bind both the “up” and “down” RBDs and contact adjacent RBDs. Shedding of S1 is reported when the RBD is captured in the “up” state and premature conversion to the post-fusion state which prevents fusion of the viral membrane with the host cell membrane. Examples include bamlanivimab (LY-CoV555) and cilgavimab (AZD1061).
- Class 3 mAbs do not block ACE2 and bind both the “up” and “down” RBDs. Contact with adjacent RBDs limits movement and can lock the RBD in a closed conformation. Examples of Class 3 Abs include sotrovimab (VIR-7831), bebtelovimab (LY-CoV1404) and imdevimab (REGN10987).
- Class 4 mAbs do not block ACE2 and bind only the “up” RBDs. Shedding of S1 is reported when the RBD is captured in the “up” state. Examples include C1C-A3, CR3022 and S304.

There are multiple mechanisms for how Abs neutralize and clear viruses. Antibodies can bind the RBD and block (directly or indirectly) binding to receptor thus preventing viral entry into the host cells [36]. Some Class 2 mAbs induce premature shedding of the S1 domain [56], thus inducing the post-fusion state and preventing fusion of the host-viral membranes. Bivalent crosslinking of the S proteins can result in steric hindrance or aggregate virions and neutralize viral entry. Finally, the Fc portion of Abs can interact with Fc gamma receptors found on myeloid and natural killer cells. These interactions are important for viral clearance brought about by engaging different receptors and inducing either Ab-dependent cell-mediated phagocytosis (ADCP), Ab-dependent cellular cytotoxicity (ADCC) or activation of the complement pathway [52]. For effective antiviral protection, a cocktail of mAbs with more than one mechanism of action might be required.

### 2.4. Escape Mutations

RNA viruses replicating via an RdRp have high rates of mutation in nature, which presents challenges for designing effective vaccines or Ab therapeutics [57,58].

Multiple structures of the S protein in complex with ACE2 are available; both SARS-CoV and SARS-CoV-2 bind to the receptor in a similar manner despite low sequence similarity in their RBM, indicating a high tolerance for mutations [18,19,43,59,60,61,62]. SARS-CoV-2 variants of concern (VOCs) have developed resistance to neutralizing Abs, including some clinical Abs [63]. The B.1.351 (Beta) VOC was found to have the largest magnitude of immune evasion upon acquiring the E484K and K417N mutations (Table 2) [64], whereas B.1.617.2 (Delta) quickly outcompeted all other circulating variants through acquisition of mutations (L452R and T478K) that enhanced transmission and pathogenicity, as well as eroded neutralizing Ab responses. The recently emerged SARS-CoV-2 Omicron variant harbors 37 amino acid substitutions in the S protein, 15 of which are in the RBD and 10 in the RBM [42,65]. Despite a significant number of mutations around the ACE2-binding site, Omicron binds to ACE2 with enhanced affinity relative to the Wuhan-Hu-1 strain and is the dominant strain circulating around the globe at the time of writing (February of 2022).

Out of the eight mAbs currently authorized by the US FDA (Table 1), six (bamlanivimab, etesevimab, casirivimab, imdevimab, cilgavimab, and tixagevimab) directly block binding of the S protein to ACE2 [42]. These mAbs are frequently used in combination to bring about maximum coverage. The second class of mAbs, represented by sotrovimab, do not block ACE2 binding, but neutralize SARS-CoV-2 by targeting non-RBM epitopes shared across many sarbecoviruses, including SARS-CoV. Comparison of the in vitro neutralizing activity of therapeutic mAbs from these two groups against Wuhan-Hu-1 S and Omicron S proteins using VSV pseudoviruses revealed that the RBM-specific mAbs lost their neutralizing activity except for the cilgavimab and tixagevimab cocktail, where there was a ~200-fold reduction in potency [14,42]. Sotrovimab was found to have 3-fold reduced potency against Omicron likely brought about by the G339D mutation (not shown in Table 2). Bebtelovimab was granted EUA by the US FDA in February 2022. Bebtelovimab binds and potently neutralizes all currently known VOCs of SARS-CoV-2 including the Omicron variant [66]. As shown in Table 2, the binding epitope of bebtelovimab is very similar to imdevimab, and the structural location of the epitope is closer to the canonical Class 3 binder, VIR-S309 [66]. 

### 2.5. Conclusions and Future Directions

The RBD is highly immunogenic but is prone to accumulate mutations. Under strong immune selection, escape mutants rapidly arise as we observe with the Omicron variant [14,42]. Further, as we prepare for emerging coronaviruses with “pandemic potential”, there is limited opportunity for having Abs with cross-reactivity to SARS-CoV-2 and other coronaviruses due to the high diversity in RBD sequences. The membrane-anchored S2 subunit, which contains the membrane fusion machinery exhibits a higher level of protein sequence conservation across coronavirus S proteins. Several teams have identified a class of S2-targeting Abs with broad reactivity towards several human betacoronaviruses from distinct subgenera, and characterized their antiviral activity, epitope and in vivo protective efficacy [52,56,68,69,70]. S2-specific mAbs can prove to be very useful, and their binding could inhibit conformational changes necessary for membrane fusion to occur. S2-specific mAbs, however, may not be sufficiently potent in viral neutralization, and enhanced Fc effector function might be necessary to achieve in vivo efficacy [52]. These mAbs can be used in combination with clinically proven neutralizing anti-SARS-CoV-2 therapies to achieve a broad neutralization spectrum across all SARS-CoV-2 variants.

## 3. Main Protease (Mpro)

### 3.1. Structural Organization and Function of Mpro

**Figure 3:**
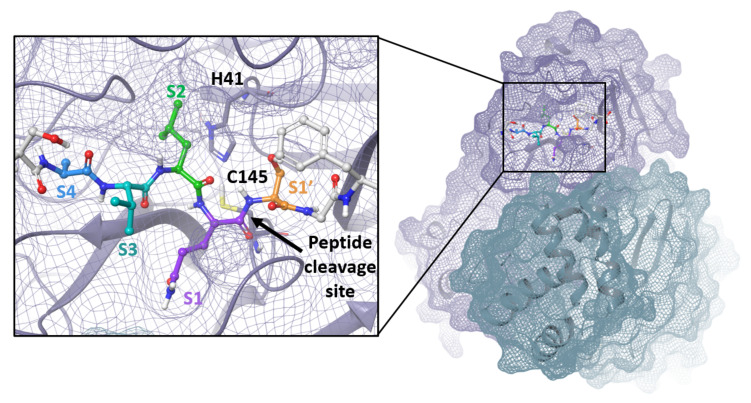
Mpro dimer and active site with peptide substrate bound. Image produced from PDB ID 7N89 with C145A mutant modeled back to cysteine [73].

**Figure 4:**
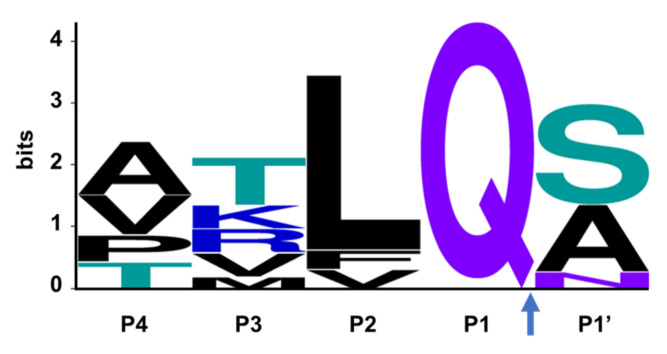
The consensus recognition sequence cleaved by SARS-CoV-2 (Uniprot code P0DTD1) Mpro. The cleavage site is marked by the blue arrow. Image generated by WebLogo [74].

The SARS-CoV-2 Mpro is a 33.8-kDa protein which is responsible for proteolytic cleavage of viral polyproteins and is essential for viral replication. X-ray analysis has revealed that two Mpro proteins associate to form a dimer which is required for catalytic activity, although it has been shown that Mpro exists as a mixture of monomer and dimer in solution [71,72]. Mpro is composed of three domains, with domains I and II forming the catalytic active site including the catalytic dyad, Cys145 and His41, and substrate-binding subsites, S1, S2, S4 and S1′ (Figure 3). Mpro recognizes and cleaves the polyproteins at 11 sites with highly conserved sequences characterized by the P1 (substrate residue N-terminal to the cleavage site) glutamine residue (Figure 4) which forms a hydrogen bond between the amide side chain carbonyl and the conserved S1 residue His163. Mpro is an attractive target for the development of small molecule antiviral therapeutics for the current pandemic as well as broad-spectrum coronavirus antivirals [71,72]. This great interest stems from the recognition that Mpro has a highly conserved active site across coronaviruses and there is no human cysteine protease with similar substrate specificity, suggesting Mpro inhibitors could be developed into broad-spectrum anti-coronavirus antivirals with high selectivity.

### 3.2. Discovery of Mpro Inhibitors

During the early stages of the COVID-19 pandemic in 2020, drug discovery organizations worldwide scoured their collections for existing drugs or candidates that could be repurposed to battle the novel coronavirus [21,22,75]. Many of the leading candidates for COVID-19 treatment originated in programs targeting the human coronaviruses SARS-CoV and MERS-CoV, in addition to unrelated rhinoviruses, enteroviruses, HIV and HCV.

**Figure 5:**
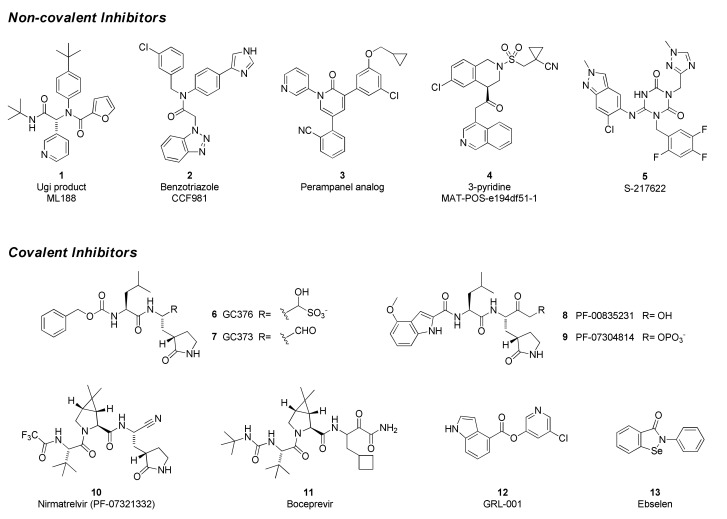
Chemical structures of representative SARS-CoV-2 Mpro inhibitors.

SARS-CoV-2 Mpro inhibitors can be classified as either *covalent*, which bear an electrophilic “warhead” that traps the catalytic cysteine to form a complex where the inhibitor is linked to the enzyme, or *non-covalent*, which do not form such an adduct (Figure 5). Many of the former are peptidomimetics that maintain a P1 glutamine-mimetic fragment to provide specificity and improve affinity, as well as a lipophilic P2 substituent. Commonly used warheads include aldehydes (or masked aldehydes), activated ketones, Michael acceptors and nitriles. Formation of the inhibitor–enzyme adduct is typically reversible and is highly stabilized by extensive hydrogen-bonding and electronic effects that mimic the transition state for protease cleavage of the enzyme substrate. In contrast, non-covalent inhibitor designs rely on hydrogen bonding, van der Waals forces, and hydrophobic interactions to create reversible binding that blocks the Mpro active site and may or may not involve the catalytic residues. It has been hypothesized that these types of drugs will have lower potential for off-target toxicities due to their lack of reactive functionalities but may suffer from weaker inhibitory activity or shorter duration of action. Non-covalent scaffolds have also served as starting points for the design of covalent inhibitors by addition of reactive groups at appropriate positions.

The Ugi class of non-covalent inhibitors originated in a high-throughput screen of the NIH sample library in search of novel SARS-CoV Mpro inhibitors in the early 2000s. The simple dipeptide structure is easily assembled using the Ugi multicomponent coupling methodology, which allowed for rapid SAR exploration. The lead compound ML 188 (**1**) was later shown to have similar and perhaps improved activity against SARS-CoV-2 Mpro (IC_50_ = 2.5 µM). The benzotriazole class was also discovered by screens targeting SARS-CoV [76,77]. In response to the COVID-19 pandemic, work on this series was refocused on SARS-CoV-2, leading to improved analogs such as CCF981 (**2**) that exhibited several orders of magnitude improvement in Mpro potency (IC_50_ = 68 nM) with submicromolar antiviral activity in infected Vero E6 cells (CPE EC_50_ = 497 nM). Perampanel, an anti-epileptic agent, was found to be a weak Mpro inhibitor through a repurposing effort that evaluated approximately 2000 known drugs [78]. Structure-guided optimization afforded a perampanel analog (**3**), which has a SARS-CoV-2 Mpro inhibitory potency of IC_50_ = 0.17 µM and antiviral EC_50_ = ~1 µM in a plaque assay in Vero E6 cells with low cytotoxicity. The isoquinoline-containing Mpro inhibitor (**4**) is a lead compound discovered through a unique crowd-sourced initiative called the COVID-19 Moonshot program. Scientists worldwide were able to contribute designs through a web portal, and donations enabled the synthesis and testing of compounds [79]. Data and x-ray structures provided by partners including the Diamond Light Source and the Weizmann Institute were made publicly available in real time. Lead compound **4** is a potent inhibitor of SARS-CoV-2 Mpro (IC_50_ = 37 nM) and demonstrates antiviral activity against several circulating variants in HeLa-ACE2 cells [80]. The consortium is hoping to advance this compound or an analog to clinical development. Shionogi has reported on S-217622 (**5**), an orally delivered Mpro inhibitor with activity against SARS-CoV-2 variants (EC_50_ = 23.9–61.7 nM, 293T-ACE2-TMPRSS2 cells), that has completed Phase 2a studies [81,82].

GC376 (**6**) is the prodrug form of a peptidomimetic drug GC373 (**7**), which was originally developed for feline coronavirus (feline infectious peritonitis virus) infections [83]. Anivive, a biotech company focused on animal healthcare, is reportedly working with partners to expand use of the compound to treat COVID-19 infections in humans [84]. The chemical structure of GC376 features a gamma-lactam as a P1 glutamine bioisostere and an aldehyde-bisulfite adduct as a latent covalent warhead. X-ray structures establish that the isobutyl sidechain occupies the lipophilic S2 site and that key hydrogen-bonding interactions are made between the inhibitor and backbone amides of Glu166, His164 and Gln189. The active form **7** exhibits moderate potency against SARS-CoV-2 Mpro (IC_50_ = 0.40 µM), similar to its level of activity against FIPV Mpro (IC_50_ = 0.49 µM) but weaker than observed with SARS-CoV (IC_50_ = 0.070 µM). Plaque assays in infected Vero E6 cells demonstrate that both compounds **6** and **7** have antiviral activity (EC_50_ = 0.92 and 1.5 µM, respectively). In a virus yield reduction assay, both compounds decreased SARS-CoV-2 titers by approximately three orders of magnitude [83].

GC376 is the prototype of a rapidly expanding class of covalent peptidomimetic Mpro inhibitors. Many publications describe related compounds, with the warhead and P3–P4 regions most frequently targeted for modification. At the beginning of the COVID-19 pandemic, Pfizer advanced PF-00835231 (**8**) and its phosphate prodrug form PF-07304814 (**9**) as intravenous (IV) drugs for early-stage infections [85,86]. The parent is potent in biochemical assays, with subnanomolar affinity for SARS-CoV-2 Mpro (K_i_ = 0.27 nM) and good activity across a broad panel of alpha- and beta-coronaviruses. Since the compound is a substrate for efflux transporters, the in vitro antiviral activity is highly dependent on the cell line and the concentration of added efflux pump inhibitor. PF-00835231 reduced viral titers in a dose-dependent fashion in SARS-CoV- and SARS-CoV-2-infected mouse models at unbound plasma C_min_ concentrations of approximately 0.7 fold of the in vitro EC_90_. Despite the poor oral pharmacokinetic profile of PF-00835231, due to the urgency of the pandemic as well as acceptable efficacy and safety of the compound, the clinical trials of PF-00835231 (as an IV administered drug) were initiated in late 2020.

An IV drug that must be administered in a clinical setting is not optimal for treating COVID-19 in the early stages of an infection when an antiviral is most likely to have maximum benefit. Therefore, an oral drug is highly desirable, and Pfizer followed PF-00835231 with a second compound that meets this criterion. Nirmatrelvir (PF-07321332, **10**) is the active component of Paxlovid, a two-drug combination that was recently granted EUA in the United States for the treatment of COVID-19 infections [87]. The second component is ritonavir, a CYP3A inhibitor that improves exposure of the antiviral compound. Structurally, nirmatrelvir contains the same glutamine-mimetic fragment as PF-00835231 but replaces the aldehyde warhead with a less reactive yet still reversible nitrile group. The remainder of the structure resembles boceprevir (**11**), an HCV protease inhibitor that has also been shown to inhibit SARS-CoV-2 Mpro. The bicyclic proline variation and trifluoroacetamide groups were necessary to improve permeability and oral absorption. Nirmatrelvir has slightly lower affinity for SARS-CoV-2 Mpro (K_i_ = 3.11 nM) than the earlier IV drug candidate **8** but maintains similar antiviral activity, with an EC_50_ = 78 nM in A549-ACE2 cells. Clinical data revealed high efficacy (up to 89% reduction in risk of hospitalization or death) in at-risk patients who began treatment within 5 days of infection [88]. Whereas GC376, nirmatrelvir and related compounds are reversible covalent inhibitors, GRL-001 (**12**) and ebselen (**13**) are representatives of a category of non-peptidic small molecules that react with the catalytic cysteine in an irreversible and time-dependent manner to produce an inactivated enzyme.

Several additional SARS-CoV-2 Mpro inhibitors have been developed for which clinical trials have been initiated or preclinical data have been presented, but their chemical structures have not been disclosed yet. Enanta has released preclinical data for EDP-235, an oral Mpro inhibitor with a biochemical IC_50_ of 5.8 nM and antiviral activity in primary human airway epithelial cells with an EC_90_ of 33 nM [89]. Cocrystal Pharma has announced plans to advance two Mpro inhibitors, CDI-988 and CDI-873, to clinical trials in 2022 [90]. Aligos has reported preclinical data for ALG-097111, which demonstrated a biochemical IC_50_ of 7 nM and antiviral EC_50_ of 200 nM (A549-ACE2 cells) [91].

### 3.3. Conclusions and Future Directions

The discovery and advancement of the first-generation SARS-CoV-2 Mpro inhibitors to clinical trials and the approval of Paxlovid for the treatment of COVID-19 infections are unprecedented in their speed, with Paxlovid demonstrating significant clinical benefit in reducing severe illness, hospitalization, and death. In the future, Mpro inhibitors that do not require pharmacokinetic boosting may enable broader utilization of these inhibitors with less risk for drug–drug interactions and use for pre- and post-exposure prophylaxis for high-risk patient populations. Previous experience with direct-acting antivirals against other viruses such as HIV and HCV suggests that combinations of drugs with different mechanisms of action may provide higher efficacy and prevent the emergence of resistance, an area of research that will become clearer as the first Mpro inhibitor sees more widespread use.

## 4. RNA-Dependent RNA Polymerase (RdRp)

### 4.1. Structural Organization and Function of RdRp

**Figure 6:**
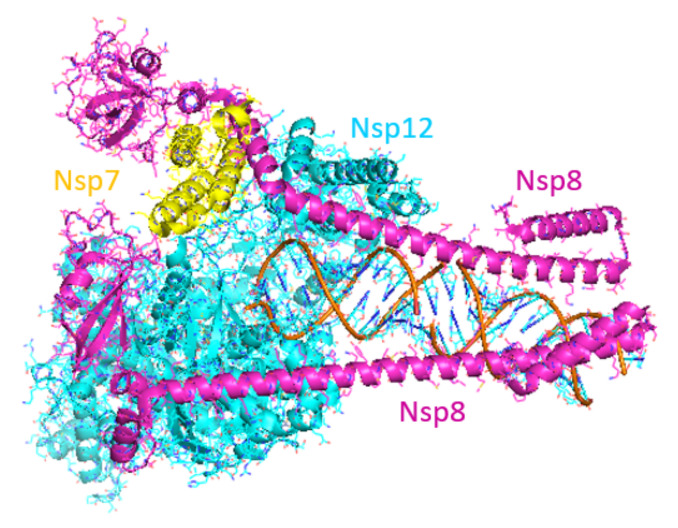
Structure of SARS-CoV-2 RdRp complex. Image produced from PDB ID 6YYT. (Nsp7: yellow; nsp8: magenta; nsp12: cyan.)

Faithful replication of the coronavirus genome is a complicated process that includes RNA synthesis, proofreading, template switching and 5′-capping, resulting in the formation of genome-length (+)-strand RNA for incorporation into newly formed virions as well as a variety of subgenomic (+)-strand RNAs that are translated into structural and accessory proteins [92]. The core SARS-CoV-2 RdRp consists of the 934 amino acid nsp12 protein, the 85 amino acid nsp7 protein and the 200 amino acid nsp8 protein. RNA synthesis occurs within the nsp12 protein but only when the nsp7 and nsp8 accessory proteins are complexed with it in a 1:1:2 stoichiometry [93,94]. Cryo-electron microscopy studies have shown that the polymerase domain of nsp12 resembles a human “right hand” consisting of fingers, thumb and palm subdomains. The two copies of nsp8 bind to nsp12 on opposing sides of the RNA-binding cleft. The C-terminal domain of one copy of nsp8 also binds to nsp7, whereas the C-terminal domain of the second nsp8 copy adopts a different fold and interacts with nsp12 directly. When long duplex RNA is present, the N-terminal domains of both nsp8 copies form long helical extensions that contact the RNA duplex product. These extensions likely act as sliding poles to enhance the processivity of the RdRp core complex and enable it to efficiently replicate the long genome of the coronaviruses (Figure 6) [94,95]. Additional proteins are thought to associate with the core RdRp complex to perform the helicase and proofreading functions.

### 4.2. Discovery of RdRp Inhibitors

**Figure 7:**
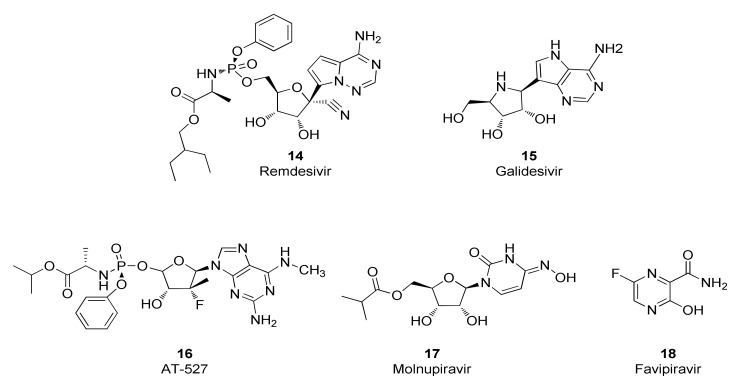
Chemical structures of representative SARS-CoV-2 RdRp inhibitors.

The SARS-CoV-2 RdRp was deemed to be an attractive drug target given the pharmaceutical industry’s success in discovering drugs, most notably nucleoside and nucleotide analogs and their prodrugs, that inhibit the RdRps for other viruses such as HIV, HBV and HCV [96,97]. Nucleos(t)ide analogs often possess inhibitory activity against the RdRps from several viral families. Indeed, drug repurposing studies identified a group of nucleos(t)ide analogs that inhibit the SARS-CoV-2 RdRp and have subsequently been investigated for their potential to elicit therapeutic benefits in humans infected with the SARS-CoV-2 virus (Figure 7). These compounds are prodrugs that require transformation to the nucleotide monophosphate level before being metabolized by host cell enzymes to the corresponding nucleotide triphosphate which is the form that is recognized by SARS-CoV-2 RdRp. Accordingly, antiviral potencies for nucleos(t)ide analogs are dependent, at least in part, by the ability of the host cells to synthesize the triphosphate form. For example, RDV (**14**) inhibits replication of the SARS-CoV-2 virus with EC_50_ values of 1 µM in Vero E6 cells [23], 0.28 µM in Calu-3 cells, 0.115 µM in H549-ACE2 cells and 0.0099 µM in primary human airway epithelial cells [24].

**Figure 8:**
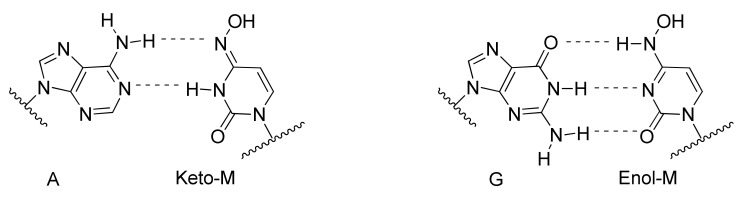
Keto-enol tautomeric equilibrium provides opportunities for molnupiravir (M) in the RNA template to form Watson–Crick hydrogen bonds with incoming ATP or GTP.

Interestingly, although these compounds belong to the nucleos(t)ide analog class, several distinct inhibitory mechanisms of action are represented. Incorporation of the RDV nucleotide into RNA by SARS-CoV-2 RdRp stops RNA synthesis after three additional nucleotides are incorporated. Further translocation and nucleotide incorporation are blocked at this point due to a steric clash between the conserved S861 side chain and the 1′-cyano group of the incorporated RDV nucleotide [98,99]. Should this translocation blockade be overcome by high NTP levels leading to full-length (−)-strand RNA containing the RDV base in several locations, a second inhibitory mechanism may come into play that includes reduced efficiency of incorporation for UTP across from template RDV, as well as for incorporation of the next correct nucleotide in the primer strand [100]. Although mechanistic studies of SARS-CoV-2 RdRp with galidesivir (**15**) have not been reported, it has been shown that nucleotide incorporation into RNA by the RdRp of HCV results in RNA chain termination after two additional NTPs are incorporated [101]. Similarly, mechanistic studies are lacking for SARS-CoV-2 RdRp inhibition by AT-527 (**16**), whose base is converted into guanine in vivo to form the active triphosphate metabolite AT-9010 [102]. However, it should be noted that the uridine analog of AT-9010, sofosbuvir, induces immediate chain termination after incorporation into RNA by SARS-CoV-2 RdRp, likely mediated by strong steric hindrance introduced by its bulky 2′-methyl group [103,104]. The mechanism of action for molnupiravir (**17**) is complex and distinct from chain-terminating nucleotides. The 4-oxime moiety of molnupiravir’s chemical structure can exist as an equilibrium of tautomeric forms—the keto form resembling uridine and the enol form resembling cytosine (Figure 8). Steady-state kinetic experiments demonstrated that molnupiravir triphosphate preferentially incorporates opposite template guanosine when incorporated into RNA by SARS-CoV-2 RdRp, thereby acting predominantly as a cytosine analog. Notably, neither this step nor incorporation of subsequent nucleotides seems to be appreciably inhibited [105,106]. In contrast, when molnupiravir’s base is present in the template, it can resemble cytosine or uridine, leading to incorporation of GTP or ATP into the opposite strand, the latter resulting in a G-to-A transition mutation if one considers the sequence G→M (molnupiravir) then M→A. C-to-U transition mutations can occur if one considers several rounds of RNA synthesis and the sequence C→G; G→M; M→A; A→U [105]. Incorporation of a guanosine nucleotide opposite the template molnupiravir base does inhibit the RdRp, but the inhibition can be overcome by high NTP concentrations [105]. Thus, molnupiravir can modestly inhibit RNA synthesis, but it also acts as a viral genome mutagenic agent. Given that molnupiravir treatment of SARS-CoV-2 virus induces significant G-to-A and C-to-U transition mutations in the viral RNA, it seems that the mutagenic effect predominates, leading to an “error catastrophe” and the eventual failure to generate replication competent viral populations [107]. Similarly, favipiravir (**18**) treatment of SARS-CoV-2-infected cells results in a significant elevation of C-to-U and G-to-A transition mutations in the viral genome, consistent with its incorporation primarily as a guanosine analog but with the ability to efficiently serve as a template base for pairing with UTP, suggesting that induction of viral mutagenesis likely plays a role in the antiviral mechanism of action for this compound [108].

Remdesivir was approved by the US FDA in October, 2020 for the treatment of COVID-19 infections in hospitalized subjects. The approval of this intravenously administered drug was based on three clinical studies, the ACTT1 study [109] and two studies sponsored by Gilead [110,111], but omitted results from a study conducted in China [112]. Shortly after approval, the results from the much larger Solidarity trial were published [113]. A meta-analysis of four of these randomized controlled trials with 7334 patients concluded that subjects treated with RDV were more likely to demonstrate recovery and were associated with higher rates of hospital discharge, but there was no significant reduction in mean time to clinical improvement or mortality [114].

Molnupiravir exhibits an in vitro antiviral EC_50_ value of 0.3 µM against SARS-CoV-2 virus when tested in Vero cells [107] as well as broad-spectrum antiviral activity against seasonal coronaviruses [115]. In preclinical models, molnupiravir inhibits SARS-CoV-2 replication in the Syrian hamster model [116,117] and mice [107] and blocks SARS-CoV-2 transmission in ferrets [118]. Given its mechanism of action as a viral mutagenesis agent, molnuprivar has been closely scrutinized for its potential to elicit DNA mutagenesis in host cells and tissues. One lab has found that molnupiravir displays host mutational activity in an animal cell culture assay [119] although molnupiravir was negative in a 28 day transgenic rodent mutagenicity study [28]. In addition, this orally administered compound was safe and well tolerated in Phase 1 studies conducted in healthy human volunteers [27]. A Phase 3 clinical study of 1408 unvaccinated participants demonstrated that molnupiravir treatment for 5 days reduced the risk of hospitalization and death by 30% [120]. Molnupiravir received EUA by the US FDA in December, 2021 for treatment of mild-to-moderate COVID-19 infections. Molnupiravir is not authorized for use in patients younger than 18 years of age because it may affect bone growth and cartilage formation and it is also important to recognize the drug may cause fetal harm when administered to pregnant individuals [28].

Galidesivir inhibits replication of SARS-CoV and MERS-CoV viruses in Vero E6 cells with EC_50_ values of 57.7 and 68.4 µM, respectively [101]. Early administration of galidesivir in a COVID-19 animal model reduced the viral burden and pathology in lung tissue [121]. A small Phase I study in COVID-19 patients demonstrated that galidesivir was safe and generally well tolerated, but it did not show signs of significant clinical benefit. Consequently, the sponsor has discontinued plans to develop galidesivir for treatment of COVID-19 [121]. AT-527 was initially developed as a drug to treat HCV infections but its EC_90_ value of 0.47 µM in a virus yield reduction assay against SARS-CoV-2 in primary human airway epithelial cells suggested potential utility for treating COVID-19 [102]. Unfortunately, AT-527 failed to meet its primary goal of reducing SARS-CoV-2 RNA at various intervals in a Phase 2 clinical trial in subjects with mild or moderate COVID-19 in the outpatient setting, leading the sponsor to update its clinical development strategy [122]. The orally administered drug favipiravir, approved to treat novel influenza in Japan, inhibits SARS-CoV-2 replication and the generation of cytopathic effects in Vero E6 cells with EC_50_ values of 207 and 118 µM, respectively [108]. Russia has approved favipiravir for treating COVID-19 infections and several other countries such as Mexico, India and Malaysia have granted EUA for this indication. A meta-analysis on clinical studies that evaluated the efficacy and safety of favipiravir as a treatment for COVID-19 found that there was a significant clinical and radiological improvement following treatment with favipiravir in comparison to the standard of care but with no significant differences on viral clearance, oxygen support requirement and side effect profiles [123].

### 4.3. Conclusions and Future Directions

Drug repurposing studies initiated at the beginning of the COVID-19 pandemic identified at least two compounds from the nucleos(t)ide analog inhibitor class, remdesivir and molnupiravir, with sufficient antiviral activity against the SARS-CoV-2 virus to merit approval or EUA by the US FDA for treating COVID-19 infections in select populations. However, both drugs have their limitations and so research is currently underway to discover new RdRp inhibitors including those from the non-nucleoside inhibitor class with improved safety and efficacy properties. Non-nucleosides are compounds that bind directly to the viral RdRp without the need for chemical transformation to the nucleotide triphosphate form and are represented in drug therapies to treat HIV and cure HCV infections [124,125]. Non-nucleosides often possess high selectivity for their viral RdRps and so it is understandable that no potent non-nucleoside inhibitors of SARS-CoV-2 RdRp emerged from early drug repurposing studies. Discovering non-nucleoside inhibitors of SARS-CoV-2 RdRp will require screening and medicinal chemistry optimization—activities at the very early stages of the drug discovery process. Fortunately, recent advances in DNA encoded libraries [126] and virtual screening [127,128] offer the ability to rapidly screen billions of molecules and should facilitate that first step on the road towards the discovery of highly selective non-nucleoside SARS-CoV-2 RdRp inhibitors.

## 5. Papain-Like Protease (PLpro)

### 5.1. Structural Organization and Functions of PLpro

**Figure 9:**
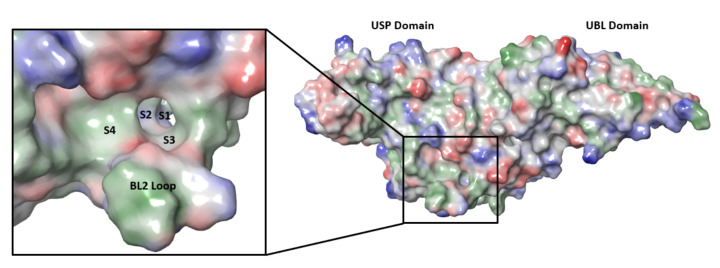
SARS-CoV-2 PLpro structure and substrate-binding site, with substrate-binding subsites of S1–S4 and the BL2 loop enlarged in the box. Image produced from PDB ID 6WUU. (UBL: ubiquitin-like domain; USP: ubiquitin-specific protease fold.)

**Figure 10:**
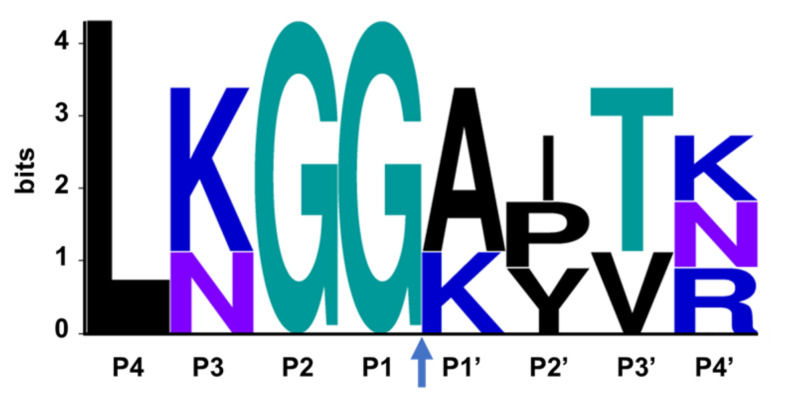
The consensus recognition sequence cleaved by SARS-CoV-2 (Uniprot code P0DTD1) PLpro. The cleavage site is marked by the blue arrow. Image generated by WebLogo.

SARS-CoV-2 nsp3 is a large multidomain protein essential for viral replication [129]. PLpro, a ~36 kDa protein, is one of the domains of nsp3 and exists as a monomer in biological settings [130]. It has a ubiquitin-like (UBL) domain at the N-terminus, and the rest of the protein has the architecture of the ubiquitin-specific protease (USP) fold (Figure 9) [130,131]. The USP fold is topologically organized into three subdomains, thumb, palm and fingers, which together form a structure resembling a right hand [132]. The SARS-CoV-2 PLpro active site contains a canonical cysteine protease catalytic triad comprised of residues Cys111, His272, and Asp286 [130,131]. It cleaves at the N terminus of the LXGG motifs (Figure 10) between nsp1, nsp2, nsp3 and nsp4, liberating the nsp1, nsp2, and nsp3 from the viral polyproteins [130,133]. Therefore, PLpro plays an essential role in the processing and maturation of the SARS-CoV-2 polyproteins.

PLpro is encoded by all coronaviruses, often in two copies, as denoted by PL1pro and PL2pro [134]. However, a number of coronaviruses, including SARS-CoV, SARS-CoV-2 and MERS-CoV, have only one copy of PLpro. The PLpro sequence between SARS-CoV-2 and SARS-CoV is highly conserved, with 83% sequence identity, and 90% sequence similarity. In contrast, the SARS-CoV-2 PLpro sequence is only 31% identical and 49% similar to that of MERS-CoV [135]. In fact, several SARS-CoV PLpro inhibitors are also known to be active against SARS-CoV-2 PLpro but not against the MERS-CoV enzyme [135,136]. In addition to the protease activity, PLpros from SARS-CoV, SARS-CoV-2 and MERS-CoV were shown in separate studies to possess deubiquitinating and deISGylating capabilities, despite the difference in the PLpro sequences among these three coronaviruses [136,137,138]. These additional enzymatic activities provide catalytic functions for cleaving ubiquitin (Ub) or ISG15 modifications from host proteins, blocking the induction of type I interferons and expression of cellular cytokines [138]. As a result, PLpro facilitates viral replication in host cells through antagonism of the host antiviral innate immune response [139,140]. Therefore, inhibition of PLpro activity can serve dual functions, controlling viral replication by targeting protease activity and restoring host immune response by targeting the deubiquitinating and deISGylating activities, making PLpro an excellent target for antiviral drug discovery.

### 5.2. Discovery of PLpro Inhibitors

While efforts to discover inhibitors for SARS-CoV-2 PLpro have been reported in the literature, there are currently no approved therapies or assets in clinical trials utilizing this mechanism. The majority of selective PLpro inhibitors target the substrate-binding pocket, although this region presents challenges for druggability (Figure 9). The S1 and S2 subsites are small and narrow to recognize the glycine residues found in PLpro substrates (Figure 10), limiting access to the active-site cysteine. The S3 and S4 regions provide a larger binding pocket but the pocket is bordered by the flexible BL2 loop.

**Figure 11:**
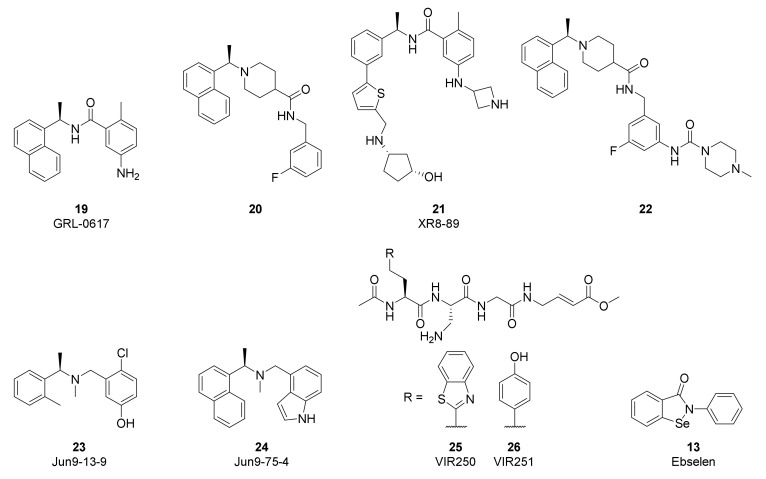
Chemical structures of representative SARS-CoV-2 PLpro inhibitors.

The high conservation of the PLpro active site between SARS-CoV-2 and SARS-CoV has accelerated the study of non-covalent inhibitors by enabling repurposing of naphthalene-based inhibitors developed to target SARS-CoV (Figure 11). Compounds such as GRL-0617 (**19**) and **20** were originally reported by Ghosh [141,142] to inhibit SARS-CoV PLpro by binding to the S3/S4 pocket and were found to have modest activity against SARS-CoV-2 (**19**: IC_50_ = 1.6 µM; **20**: IC_50_ = 2.6 µM) [135,143,144]. Efforts to improve the activity of these first-generation compounds have often grown the inhibitors to target additional interactions. Xiong and coworkers improved potency by converting the naphthalene of GRL-0617 to a substituted 2-phenylthiophene in XR8-89 (**21**, IC_50_ = 0.11 µM) in order to reach further down the BL2 groove, while also appending an azetidine to the aniline to interact with Glu167 [143]. Tan and coworkers used compound **20** as a starting point and enhanced activity by attaching the piperazine urea in **22** (IC_50_ = 0.44 µM) to extend around the BL2 loop [144]. A high-throughput screen by Wang identified dibenzylamine hits such as Jun9-13-9 (**23**, IC_50_ = 6.7 µM) that during optimization converged with the earlier naphthalene inhibitors to yield more potent compounds such as Jun9-75-4 (**24**, IC_50_ = 0.62 µM) [145]. While activity gains have recently been made in this class of non-covalent PLpro inhibitors, further improvements in antiviral potency will be required to generate candidates with clinical efficacy.

Efforts to produce covalent SARS-CoV-2 PLpro inhibitors have also been described. Olsen and coworkers used a combinatorial library of fluorogenic tetrapeptide substrates to identify the optimal peptide sequences for PLpro reactivity [146]. Replacement of the chromophore with an α,β-unsaturated ester warhead provided inhibitors VIR250 (**25**) and VIR251 (**26**). Co-crystal structures highlighted the key interactions with the protease and verified the covalent inhibition mechanism. Li and coworkers used **19** as the starting point for their covalent inhibitor and appended a sulfonium-tethered peptide to generate a peptide drug conjugate [147]. Both strategies incorporated the glycine residues of the PLpro substrates to navigate the S1 and S2 sites. Further advancements in covalent strategies will need to overcome the modest activity and challenging peptidic properties of these early compounds.

High-throughput screens of known bioactive compounds have led to reports that a variety of other classes of compounds inhibit SARS-CoV-2 PLpro. Organoselenium compounds such as Ebselen (**13**) were reported to inhibit PLpro [148], but others have found these act as non-selective modifiers of cysteine, including the cysteine at the active site of PLpro [149]. Validation studies of reported quinone and nucleoside hits also suggest these classes of compounds are non-specific inhibitors [150]. The limited antiviral activity and poor selectivity of these hits are significant challenges to advancing them into useful tools and highlight the importance of confirming PLpro screening results across multiple assays.

### 5.3. Conclusions and Future Directions

SARS-CoV-2 PLpro is a multifunctional enzyme with protease, deubiquitinase, and deISGylating activities, with the latter two activities involved in blocking the expression of type I interferons in the infected cells. In principle, targeting the activities of PLpro may not only inhibit viral replication but also block the viral-mediated evasion of host innate immunity. One recent report presented data that support this hypothesis: GRL-0617 could inhibit SARS-CoV-2 replication as well as maintain the antiviral interferon signaling in the infected cells [136]. The discovery of potent PLpro inhibitors has been challenging due to the apparent lack of well-defined binding pockets at its substrate-binding pocket. However, the growing number of structural studies conducted with PLpro to identify potential binding pockets and the increasing interest in optimizing known PLpro inhibitors based on structural data would certainly facilitate the understanding of the druggability of PLpro and advance the drug discovery for this antiviral target.

## 6. Summary

In this review, we discuss the current status and future strategies for the discovery of small- and large-molecule antiviral therapeutics for COVID-19 and, potentially, emerging coronaviruses. There is an increasing number of inhibitors reported to have anti-SARS-CoV-2 activity in vitro and in vivo. Several of these inhibitors [e.g., some Mpro or RdRp inhibitors, and at least two examples of anti-S mAbs (sotrovimab and bebtelovimab)] have also been shown to be efficacious for a reasonably wide spectrum of coronaviruses and/or COVID-19 variants [87,98,99,151]. This is not surprising due to the high protein sequence conservation among coronaviruses and variants at the catalytic sites and substrate-binding sites of Mpro and RdRp, as well as parts of the S protein that are important for viral entry. PLpro protein sequence is less conserved among coronaviruses when compared to those of Mpro and RdRp. The breadth of the antiviral spectrum of an optimized PLpro inhibitor remains to be determined. This review has focused on small molecules and mAbs that directly target viral proteins. In addition to these drug modalities, other therapeutic approaches (e.g., CRISPR, RNAi, or antisense oligonucleotide) may also prove to be useful for COVID-19 therapies.

Resistance generation during antiviral therapy can occur during treatment for chronic viral infections such as HIV and HCV. With the short duration of COVID-19 antiviral therapy, development of drug resistance to small molecule inhibitors may not be an issue. Should drug resistance become a concern, the availability of SARS-CoV-2 inhibitors targeting different viral proteins will allow combination therapy to minimize the generation of resistance.

Drug discovery is progressing at lightning speed to address the urgent need of the COVID-19 crisis. We bear witness to unprecedented partnerships among industry, academics and/or government to join forces in the discovery and development of COVID-19 drugs. In addition, the basic research, drug discovery and clinical development frameworks established by decades of antiviral research on human pathogenic viruses enable investigators to quickly apply prior knowledge and cutting-edge technology to discover treatments for COVID-19 infections. Recognizing the importance of antivirals, the US government has pledged to support a new initiative called the Antiviral Program for Pandemics by committing $3.2 billion in funding for research on COVID-19 antiviral drug development, as well as new drugs for viruses that could cause future pandemics [152]. With the availability of several validated and druggable targets for SARS-CoV-2, as well as the unprecedented partnerships and resources dedicated to COVID-19 drug discovery, we expect that a rich pipeline of COVID-19 drugs will emerge that will become important weapons to tackle COVID-19 and future coronaviruses with pandemic risk.

## Figures and Tables

**Table 1:** Antiviral drugs for the treatment of COVID-19 infections in the US.

COVID-19 Drug	Viral Target	Drug Modality	Delivery	Approval Status	Discovery Approach
Sotrovimab	Spike	Biologic	IV	EUA ^1^	Developed for SARS-CoV-2
Bebtelovimab	Spike	Biologic	IV	EUA ^1^	Developed for SARS-CoV-2
Tixagevimab + Cilgavimab	Spike	Biologic	IM	EUA ^2^	Developed for SARS-CoV-2
Bamlanivimab + Etesevimab	Spike	Biologic	IV	EUA ^1,3^	Developed for SARS-CoV-2
Casirivimab + Imdevimab	Spike	Biologic	IV/SubQ	EUA ^1,3^	Developed for SARS-CoV-2
Remdesivir	RdRp	Small molecule	IV	Approved	Repurposed Ebola inhibitor
Molnupiravir	RdRp	Small molecule	Oral	EUA	Repurposed VEEV inhibitor
Paxlovid (Nirmatrelvir + Ritonavir)	Mpro	Small molecule	Oral	EUA	Nirmatrelvir designed for SARS-CoV-2; ritonavir used as a PK enhancer

^1^ For post-exposure treatment of COVID-19. ^2^ For pre-exposure prophylaxis of COVID-19 in special populations. ^3^ Use limited by the FDA in January 2022 to treat COVID-19 due to the Omicron variant. IV: intravenous; EUA: emergency use authorization; IM: intramuscular; SubQ: subcutaneous; RdRp: RNA-dependent RNA polymerase; VEEV: Venezuelan equine encephalitis virus; Mpro: main protease; PK: pharmacokinetic.

**Table 2:** Spike RBD sequence of SARS-CoV-2 Wuhan-Hu-1 with highlighted footprints of ACE2 (pale blue), variants of concern Alpha, Beta, Gamma, Delta and Omicron with positions of mutations (peach), and 8 mAbs with binding epitopes on the RBM (pale green) [42,66,67]. (AA#: amino acid numbering of the spike protein.).

AA#	ACE2	Alpha	Beta	Gamma	Delta (+)	Omicron	Etesevimab	Bamlanivimab	Cilgavimab	Tixagevimab	Casirivimab	Imdevimab	Regdanvimab	Bebtelovimab
417	K		N	T	N	N								
438	S													
439	N													
440	N					K								
441	L													
442	D													
443	S													
444	K													
445	V													
446	G					S								
447	G													
448	N													
449	Y													
450	N													
451	Y													
452	L				R									
453	Y													
454	R													
455	L													
456	F													
457	R													
458	K													
459	S													
460	N													
461-469														
470	T													
471	E													
472	I													
473	Y													
474	Q													
475	A													
476	G													
477	S					N								
478	T				K	K								
479	P													
480	C													
481	N													
482	G													
483	V													
484	E		K	K		A								
485	G													
486	F													
487	N													
488	C													
489	Y													
490	F													
491	P													
492	L													
493	Q					K								
494	S													
495	Y													
496	G					S								
497	F													
498	Q					R								
499	P													
500	T													
501	N	Y	Y	Y		Y								
502	G													
503	V													
504	G													
505	Y					H								
